# Imprinted high-*Q* polymer micro-ring resonator array for high-resolution photoacoustic tomography

**DOI:** 10.29026/oea.2026.250215

**Published:** 2026-03-30

**Authors:** Hyeonwoo Kim, Wei-Kuan Lin, Linyu Ni, Mohammad Ali, Xueding Wang, Guan Xu, L. Jay Guo

**Affiliations:** 1 Department of Electrical Engineering and Computer Sciences, University of Michigan, Ann Arbor 48109, USA; 2 Department of Biomedical Engineering, University of Michigan, Ann Arbor 48109, USA; 3 Department of Radiology, University of Michigan, Ann Arbor 48109, USA; 4 Department of Ophthalmology and Visual Sciences, University of Michigan, Ann Arbor 48105, USA.

**Keywords:** nanoimprint lithography, micro-ring resonator array, high-quality factor, photoacoustic tomography

## Abstract

We present the first demonstration of a polymer-based micro-ring resonator array with over 40 elements fabricated using nanoimprint lithography (NIL), and its application for photoacoustic tomography imaging. By precisely varying the radius of each micro-ring at the nanometer scale and maintaining its high-quality factor, we achieved distinguishable resonances within a free spectral range of only 2.19 nm. The micro-ring array was used to perform photoacoustic tomography (PAT), achieving lateral and axial resolutions of 40 μm and 38 μm, respectively, and an ultrasound response bandwidth of 173.5 MHz, and a noise equivalent pressure (NEP) of 13.5 Pa. PAT images of *ex vivo* mouse prostates were acquired by the micro-ring array, showing a high correlation with biological tissue structures and regions of blood vessels. Further spectral analysis of the PAT images can differentiate the prostate cancers from the normal prostates. This work highlights the significant advantages of using NIL-fabricated micro-ring resonator arrays made from polymer materials, demonstrating their potential for advanced applications in biomedical imaging as well as optical communications and integrated photonic systems.

## 1 Introduction

**Fig. 1 |:**
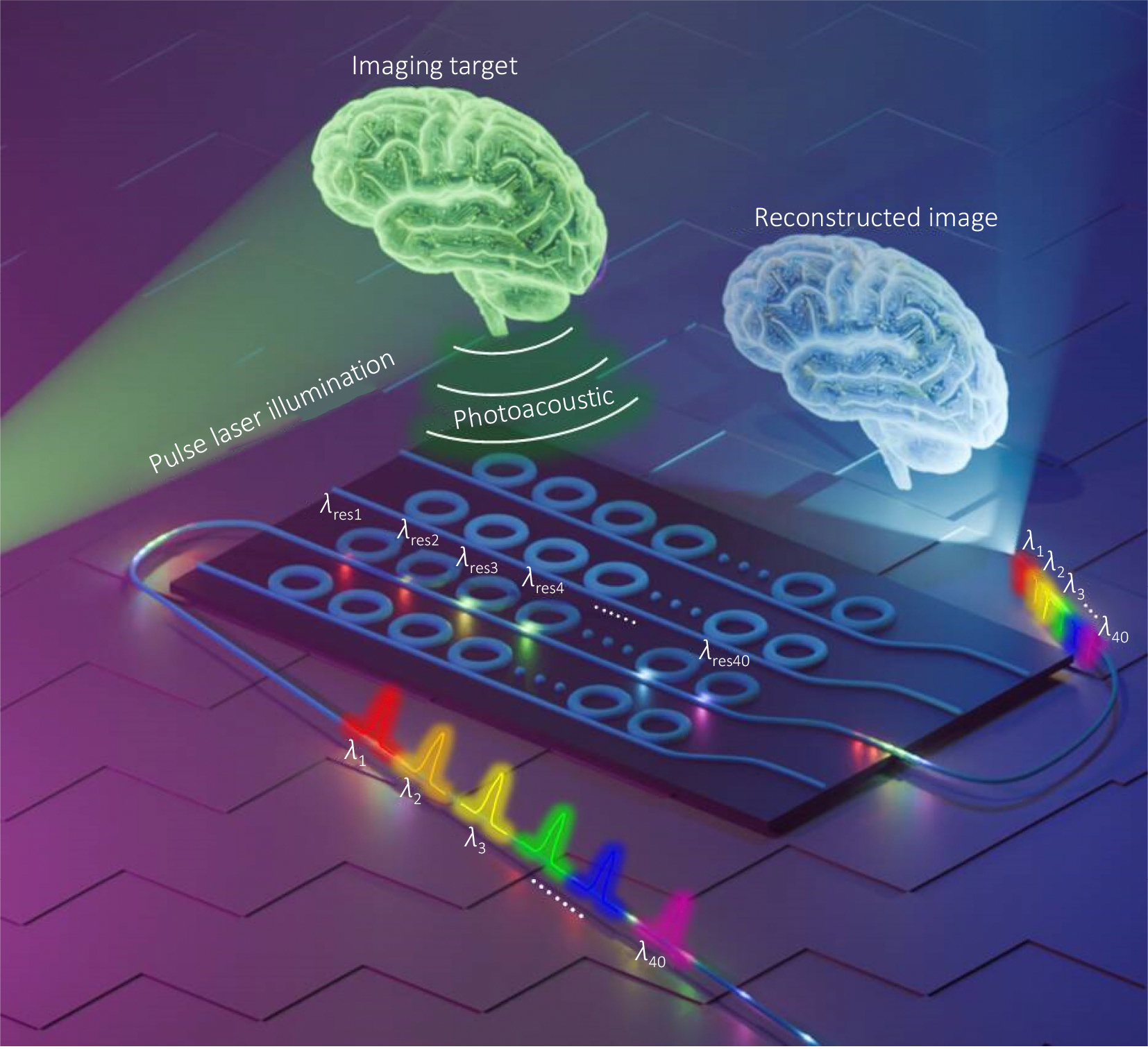
Conceptual rendering of a micro-ring resonator array that each has different resonant wavelength.

Micro-ring resonator arrays are one of the important integrated optical elements due to their exceptional capability to process multiplexed signals, which is essential for advancing numerous high-tech applications^1−3^. By precisely tuning the radius of each micro-ring at the nanometer scale, as illustrated in Fig. 1, these arrays can generate distinct resonant wavelengths within a narrow wavelength range. This precision allows for dense wavelength division multiplexing, which is pivotal in increasing the capacity and speed of optical communication systems^4,5^. Moreover, the ability to control resonances at fine scales of tens of picometers opens up opportunities for developing more efficient and compact integrated photonic circuits, impacting fields ranging from telecommunications and optical computing^2,3,6^ to photoacoustic tomography (PAT) imaging^1,7,8^.

Based on these studies, micro-ring resonators have been extensively explored as add/drop filters, lasers, modulators^9−11^, and polymer micro-rings have been exploited for sensors^12,13^, and as photoacoustic imaging detectors^14,15^ due to their high acoustic sensitivity^16^, broadband response^14^, small size, and low electrical noise. Taking advantage of these properties of micro-ring resonators, optical resolution photoacoustic microscopy utilizing single element micro-ring resonators^17^ demonstrated superior spatial resolution compared to those using traditional ultrasound transducers^18−20^. Furthermore, PAT with deep penetration achieved by mechanically translating a single micro-ring resonator^18^ or using multiple micro-ring resonators were demonstrated^1^. However, they are either limited by the need to translate the micro-ring sensor or essentially still performing photoacoustic microscopy by averaging the signals from multiple micro-rings^1^.

Developing a micro-ring resonator array with a large number of independent elements is crucial for achieving high-speed PAT imaging. However, realizing such arrays presents several technical challenges. First and foremost, the fabrication process must precisely control the radius of each ring at the nanometer scale to ensure distinct resonance from each micro-ring resonator within a very narrow free-spectral range (FSR). Furthermore, achieving high-quality factors in micro-ring resonators is essential so that the resonances are separated from each other, and the dense resonances can be clearly distinguished and indexed. Lastly, a rapid and accurate signal processing system is critical for reading signals from each individual ring and effectively controlling the array for PAT purposes.

In this work, we demonstrated the micro-ring resonator array featuring over 40 different resonant wavelengths within a free spectral range of 2.19 nm, achieved by the Nanoimprinting method. In addition, we studied a way to control a tunable input laser system to precisely read out the output signals from the resonances of micro-rings. By using this control algorithm, we demonstrated an application of the micro-ring array for PAT that requires high-frequency and high-speed acquisition of acoustic signals generated in biological tissues illuminated by nano-second laser pulses. Our PAT system integrating the micro-ring array demonstrated lateral and axial resolution under 40 μm, and showed the ability to assess the microstructure of tissue components in *ex vivo* mouse prostates, such as hemoglobin contents and prostate gland architecture.

## 2 Method

The fabrication challenges of the polymer micro-ring array are two folds. First, smooth surfaces are required to ensure low scattering loss and high-quality factor resonance^21^. Second, high adequate precision is needed to precisely delineate the resonance of the array within a very small wavelength range. Because the polymers have much lower refractive index than their inorganic counterparts commonly used for constructing the micro-ring arrays, e.g. Si_3_N_4_ and Si, high-quality factor polymer micro-ring resonators must be made with much larger sizes (e.g. diameter of 60–100 μm) to minimize the radiation or bending loss of the ring waveguide. This in turn makes the FSR of the resonances, i.e. the wavelength spacing between neighboring resonances, much smaller. To fit tens of micro-ring resonances within such a tiny FSR, the diameter variation between the neighboring micro-rings is on the order of ~1 nm, while the surface roughness of the micro-ring waveguide should be controlled down to 10 nm to minimize light scattering loss. To ensure the fabrication quality of micro-ring resonator array for achieving a high-quality factor in a narrow free spectra range, several state-of-the-art fabrication methods are explored for micro-ring arrays. Traditional electron beam lithography (EBL) has been used for the fabrication of small or intricate structures, and for making micro-rings using inorganic materials. However, this method is costly and therefore limited to small-scale production. Moreover, it cannot be used directly to pattern the polymer micro-rings due to the requirement of low material absorption loss needed for high-quality factor resonances^19,22^. Recently, two-photon lithography (TPL), also known as two-photon polymerization^23,24^, has emerged as a promising new method for fabricating micro-ring resonators. TPL can enable true 3D structuring with high resolution and versatility, capable of fabricating structures that are difficult or impossible to make with other methods. However, this method still faces challenges in the throughput of fabrication, low-quality factor, and surface roughness control of structures^25^.

Unlike other lithography methods, nanoimprinting lithography (NIL) can achieve superior patterning resolution for micro/nano structures by mechanically deforming the polymer materials^22,26−29^. This method, first demonstrated 30 years ago^29^, is based on replicating the pattern faithfully from that on an imprinting mold. NIL renders a high-throughput, high-speed patterning, high resolution down to a few nanometers, low surface roughness, and cost-effective technique for precise structures. We have shown previously that NIL is capable of fabricating high-quality factor polymer micro-ring resonators, which were used for ultrasound detection^16,17^. However, it is not certain whether the technique can offer the precision in reproducing ~1 nm differences in the diameter of an array of micro-rings while preserving low surface roughness.

**Fig. 2 |:**
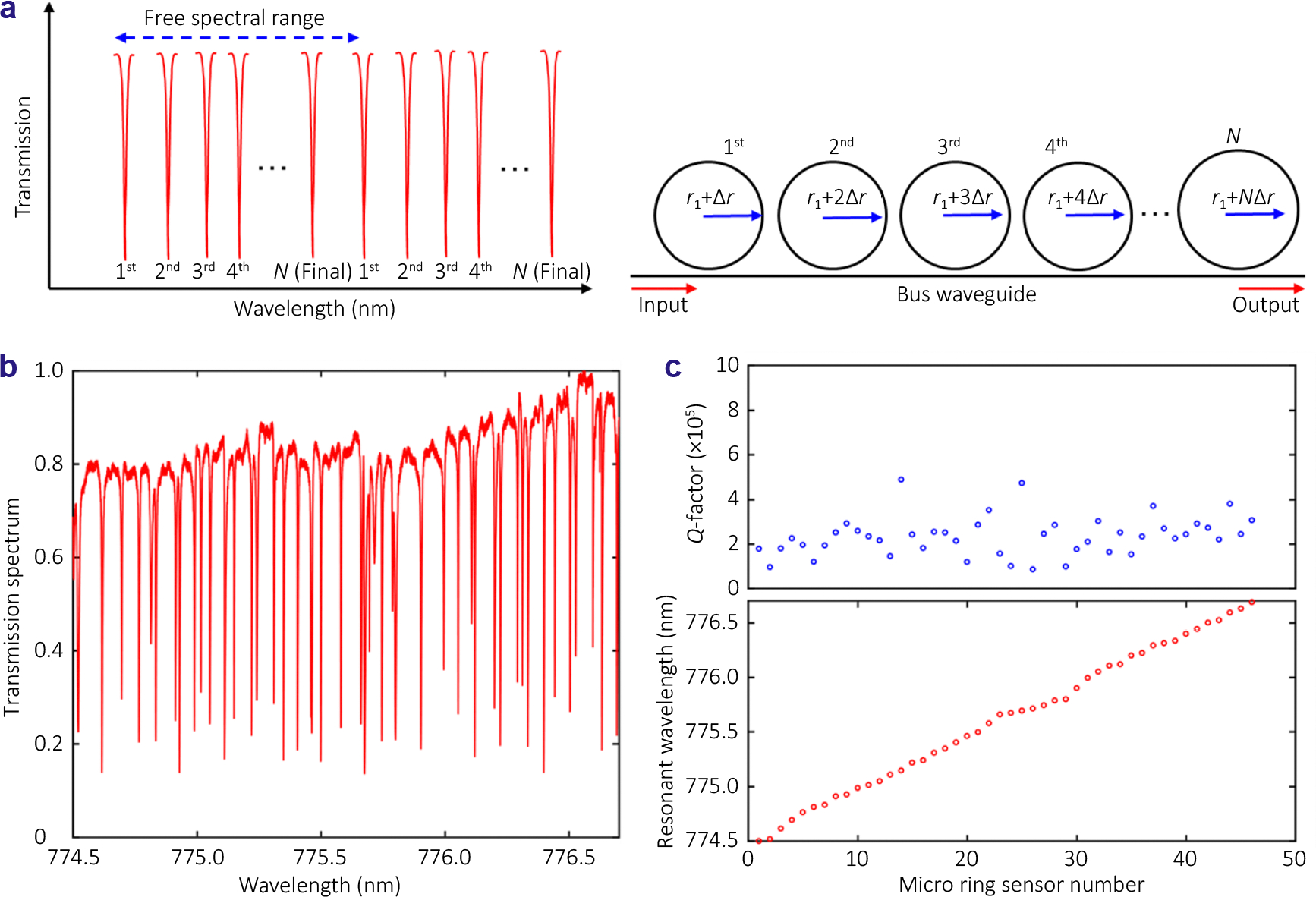
(a) Schematic diagram of the proposed micro-ring resonator array. (b) Measured transmittance of 50 elements of micro-ring array. (c) Quality factor of each ring (top) and sequence information (bottom).

To understand this requirement, Fig. 2(a) illustrates a micro-ring resonator array used to create dense wavelength division within a narrow free spectral range. The array device consists of a bus waveguide and multiple micro-ring resonators, with each ring having a different radius to produce a distinct resonant wavelength within the spectral range. This is achieved by gradually varying the ring radius with a radius difference (Δr), as described by the following equation:

(1) $$\Delta r=\frac{\lambda }{2\pi n_{eff}N}$$

where λ is a center wavelength, $n_{eff}$ is an effective refractive index, and N is the number of micro-rings.

The key parameter of a micro-ring resonator array is the ring radius (*r*_1_), as it influences both the quality factor and the free spectral range. The quality factor is affected by the scattering, radial, and absorption losses. In particular, the radial loss decreases as the ring radius increases, which enables a higher quality factor. However, the free spectral range, which is inversely proportional to the ring radius, narrows as the radius increases. Therefore, to maintain distinct resonances within the free spectral range, the selection of ring radius is constrained by the properties of the waveguide material. In our study, we selected polystyrene (*n*=1.577 @ 780 nm) as the waveguide core for 20, 40 and 50 elements of micro-ring resonator array. In the case of 50 elements, we designed micro-rings with a radius of 30 μm, in this case a radius difference of 1.57 nm between adjacent rings needs to be realized.

Figure 2(b) showcases the measured transmission spectrum of 50 elements micro-ring resonator array, of 46 highly narrow resonance dips within a tight 2.19 nm free spectral range. Most of the resonance dips correspond to individual micro-rings, as the fabricated device generally follows the design rule of varying ring radius. However, due to fabrication inaccuracies, some overlapped resonance dips or a non-sequential order of resonances occurred. To verify which resonance corresponds to each micro-ring, we recorded a video of the micro-ring array while scanning the tunable laser connected to the device via an optical fiber. When a micro-ring is in resonance, it scatters light due to enhanced light intensity in this micro-ring. By comparing the video footage with the recorded spectrum, we can correspond each resonance with a specific micro-ring. Using this information, we plotted the resonance wavelengths as a function of the corresponding micro-rings, as shown in Fig. 2(c) (bottom). The plot indicates that most rings have their own resonances within the free spectral range, although some rings did not follow the design rules attributed to the fabrication imperfection. Figure 2(c) (top) shows the quality factor of each micro-ring, with an average value of 2.34×10^5^.

**Fig. 3 |:**
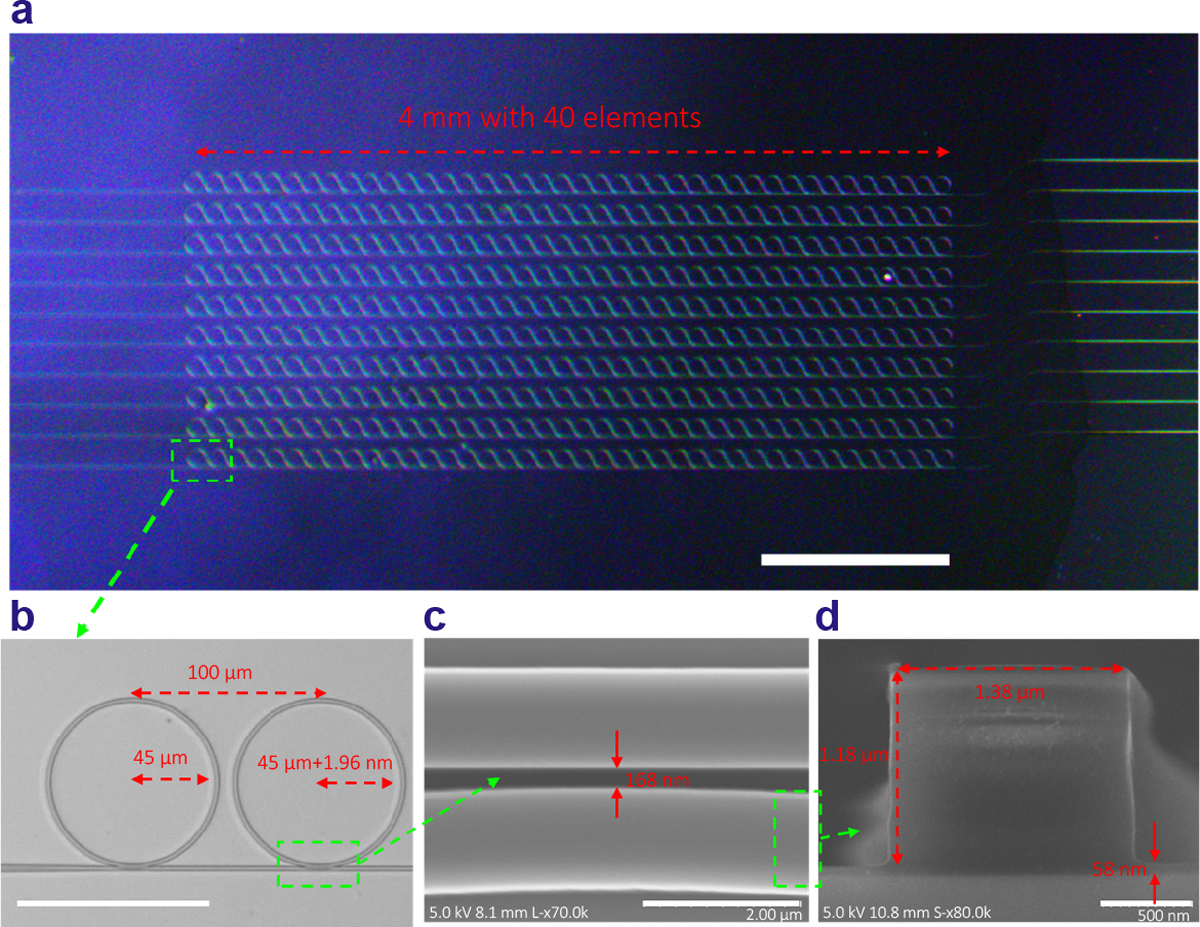
Photographs and SEM images of fabricated micro-ring resonator arrays using NIL method. (a) Photograph of micro-ring arrays under microscope with several lines with different gap sizes between the micro-rings and the bus waveguides (1 mm scale bar). (b) Photograph of two micro-ring resonators and a bus waveguide (100 μm scale bar). (c) SEM images of the gap between the bus waveguide and the micro-ring and (d) the cross section of the bus waveguide.

To achieve the required 1.57 nm difference between neighboring micro-rings, we utilized NIL to fabricate microring resonator arrays. A high-quality silicon mold was fabricated using EBL and RIE. A silicon wafer with a 2 μm SiO_2_ under cladding layer was employed to minimize light leakage from the polystyrene waveguide core to the silicon substrate, while reactive ion etching was used to reduce the residual layer after NIL to less than 100 nm. This minimizes radiation loss and maintains high quality factors. Figure 3(a) illustrates the imprinted array of 40 elements with varying gaps from 100 nm to 400 nm between the bus waveguide and the micro-rings, optimizing the coupling efficiency for device performance. Detailed fabrication processes are outlined in Supplementary Section 1.

Figure 3(a) through 3D showcase the excellent capabilities of NIL in precisely fabricating the micro-ring resonator array with 40 elements, where the ring radius increases from 45 μm to 45.076 μm in ~2 nm increments. Figure 3(b) shows that the spacing between adjacent micro-rings is 100 μm—such close element spacing is to ensure high-resolution when the array is used in ultrasound signal detection for high-quality PAT imaging. Figure 3(c) shows the gap between the bus waveguide and the micro-ring of 168 nm, which controls the light coupling. The cross-section seen in Fig. 3(d) reveals a smooth waveguide surface with a height of 1.18 μm, a width of 1.38 μm, and a residual layer of 58 nm. Additionally, the micro-ring resonator arrays are over-coated with a PDMS layer as cladding and wrapped in low-density polyethylene film serving as protection. This structure enhances spectral stability when exposed to various liquids or tissues by maintaining consistent effective refractive indices for the waveguide and cladding layer^19^.

## 3 Results

One main advantage of our developed micro-ring array is its ability to capture and reconstruct PAT images without need of translating either the micro-ring device or the imaging object. This is enabled by the array’s large field of view and independently operating micro-ring elements. Ultrasound-to-light signal transduction is achieved through precise aligning the tunable laser’s wavelength to the slope of each resonance wavelength. However, several challenges exist, such as maintaining spectral stability in unstable environments and addressing overlapping and non-sequential resonant wavelengths. These issues can be addressed by utilizing sequential information as shown in Fig. 2(c) and implementing an automated tracking algorithm to mitigate any spectrum shift caused by temperature and pressure variations. PAT image reconstruction is facilitated by the delay-and-sum (DAS) method, which combines the received photoacoustic signals from all the micro-rings. Further details on the methodologies of the tracking algorithm are in the Supplementary Section 3.

**Fig. 4 |:**
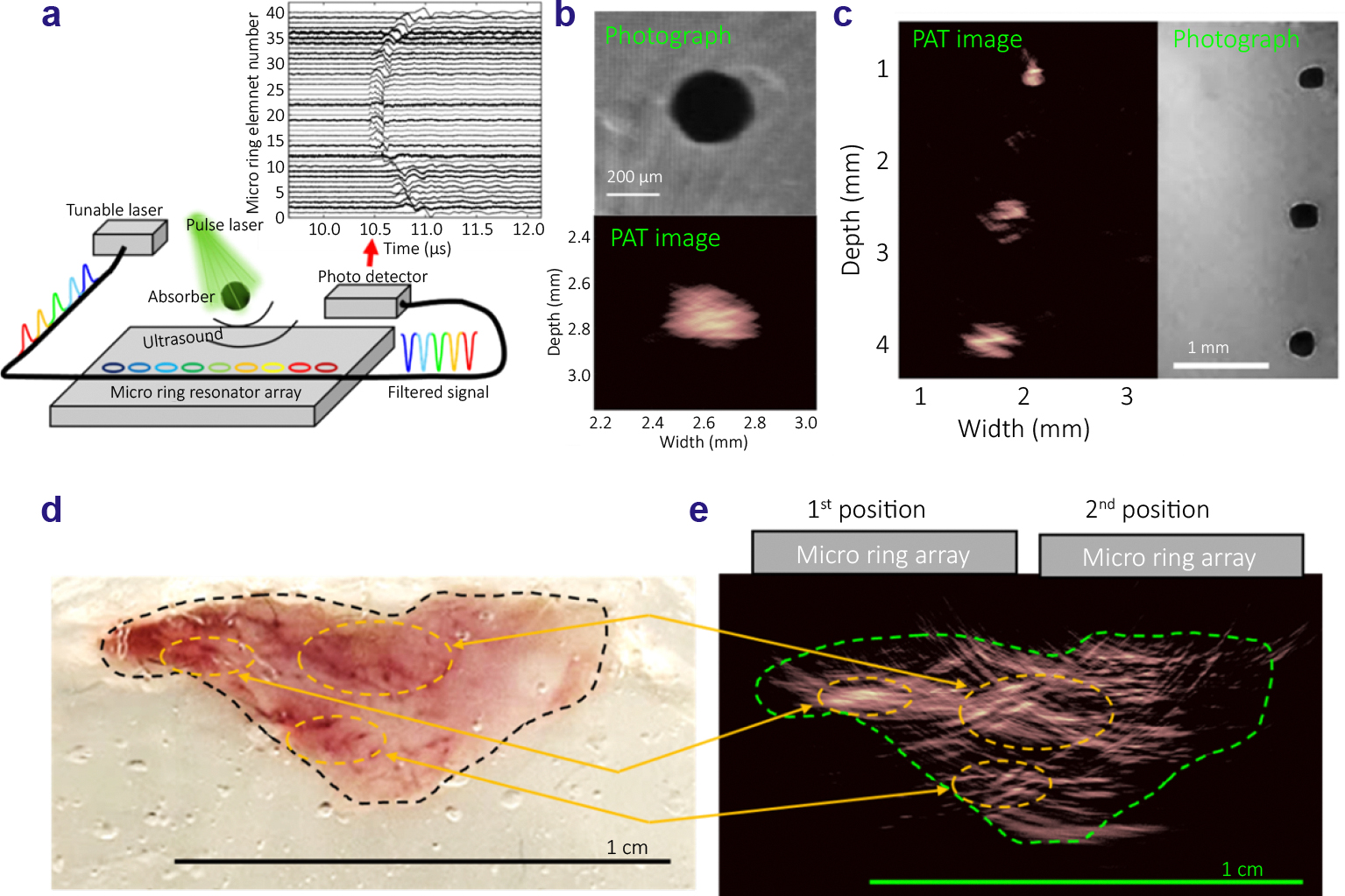
(a) Experimental configuration of PAT of a single black sphere. The inset figure shows the measured photoacoustic signals of the black sphere captured by the 40 elements of the micro-ring array. (b) Reconstructed image (bottom) from the acquired PAT signals and photograph (top) of the black sphere. (c) Photograph of multiple black spheres (right) and the reconstructed PAT image acquired by the micro-ring array (left). (d) Photograph of half of a mouse prostate and (e) the reconstructed PAT image of the prostate tissue acquired by the micro-ring array.

Figure 4(a) depicts the experimental setup for PAT imaging using a 40-element micro-ring resonator array to acquire photoacoustic signals. In this setup, a black sphere, embedded in gelatin (Sigma-Aldrich, Porcine skin), is aligned with the array and positioned approximately 2.6 mm from its center. The photoacoustic signals are generated by illuminating the black sphere with the pulsed light from a Nd:YAG pumped OPO (Surlite I-20, Amplitude), causing the target to emit acoustic signals upon absorbing the laser light.

The inset figure presents the photoacoustic signals captured by all 40 elements of the array. Each A-line signal demonstrates that, when the tunable laser’s wavelength is precisely aligned with each resonator’s slope, the micro-rings can successfully acquire photoacoustic signals with minimal noise after 20 times of signal averaging. Notably, the arc shaped delay curve clearly represents the time-of-flight of the photoacoustic signal traveling from the black sphere to each micro-ring resonator, which is used to accurately capture spatial information.

The micro-ring resonator array enables high-resolution PAT imaging, as demonstrated in Fig. 4(b). By employing the DAS method, the reconstructed PAT image reveals that a black sphere is positioned at a depth of 2.6 mm from the array, with a width of 278 μm. Further showcasing the system’s capability, Fig. 4(c) (right) presents a photograph of an imaging target comprising three black microspheres at varying depths. Figure 4(c) (left) presents the reconstructed PAT image, highlighting the system’s ability to reliably capture photoacoustic images without need to translate the target or the micro-ring device.

We further investigated the image quality of PAT using mouse prostates *ex vivo*. Note that we imaged only half of the prostate which was still larger than the width of microring array. In this study, we captured the photoacoustic signals by locating the array device at the two locations marked as 1^st^ and 2^nd^ positions in Fig. 4(e). The distance between the micro-ring array and the prostate tissue was 3.4 mm. In this way, two PAT images were acquired to cover the unique geometries of both the main and side lobes of the mouse prostate, as illustrated in Fig. 4(d). Figure 4(e) shows the merged image from the PAT images acquired at 1^st^ and 2^nd^ positions after image reconstruction. The green contour outlines the mouse prostate, showing a good match between the reconstructed image and the tissue sample profile. Additionally, since the photoacoustic signals at 532 nm laser wavelength primarily arise from the blood vessels in the mouse prostate, the bright regions in the reconstructed image highlight the areas with more blood vessels, as marked by the arrows and circles at the corresponding locations.

The axial resolution of photoacoustic imaging is determined by the detector response bandwidth^14^. To characterize the receiving bandwidth of the micro-ring resonator array, we conducted measurements following the established methods^14^. Broadband photoacoustic signals were generated by illuminating a 100 nm thick gold film with 532 nm wavelength laser pulses with a pulse duration of 6 ns, and detected by the micro-ring array in water. The 6 ns laser pulse duration leads to a photoacoustic signal bandwidth of 88.4 MHz. When assessing the receiving bandwidth of the micro-rings, the effect of this 88.4 MHz bandwidth can be removed by deconvolution. Detailed methods are described in the Supplemantary information of Section 2.

**Fig. 5 |:**
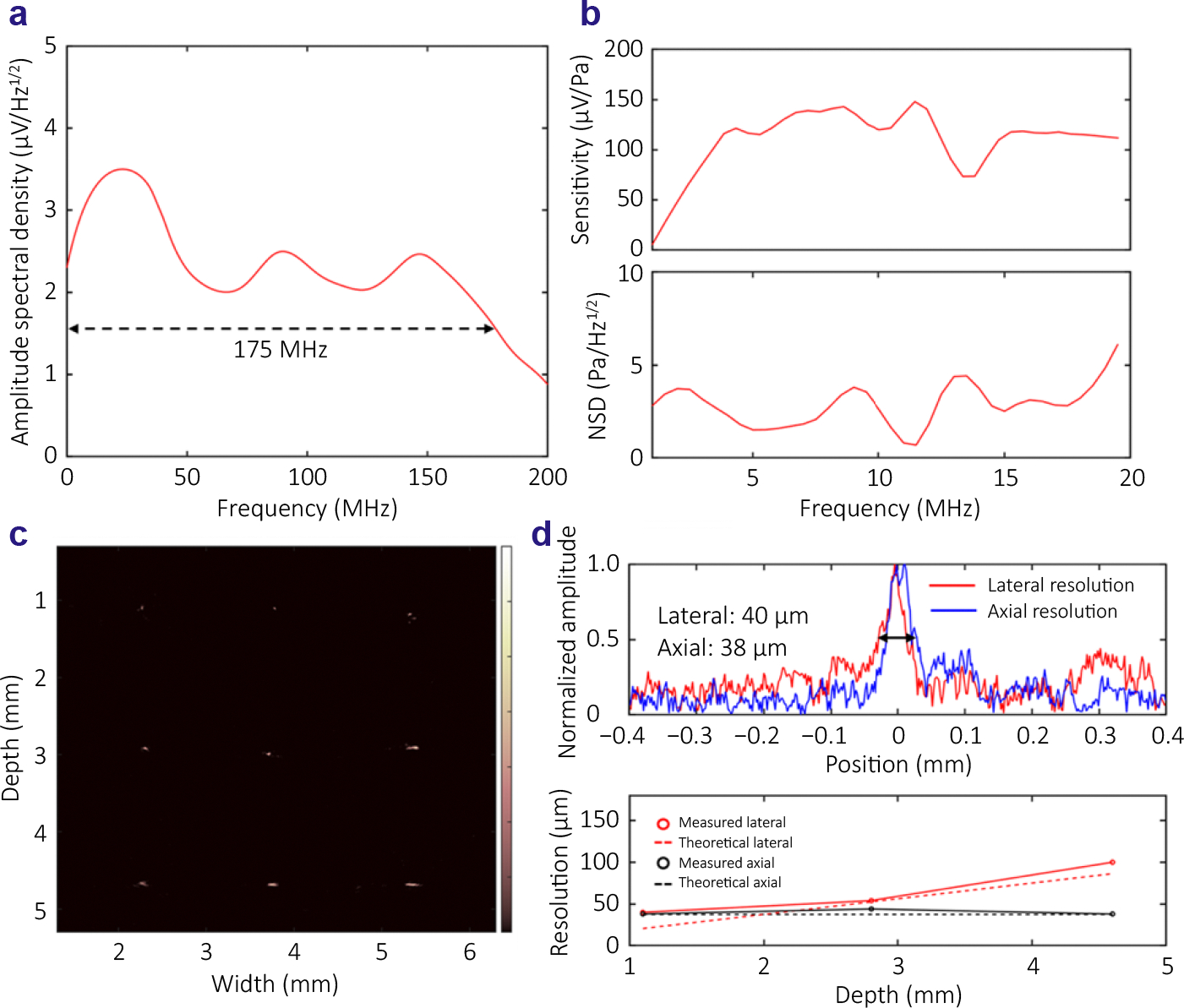
(a) Measured amplitude spectral density of a micro-ring using a broadband acoustic source. (b) Measured receiving sensitivity of the micro-ring (top) and NEP spectral density (bottom) using a calibrated hydrophone and a 10 MHz transducer. (c) Combined PAT image showing the acoustic point source placed at 3 by 3 different spot locations. (d) Measured lateral and axial resolution at the 1.1 mm depth along the center of the micro-ring array (top), and the measured lateral and axial resolutions at different depths along the center vs. the theoretical values (bottom).

Figure 5(a) illustrates the spectral frequency density of the broadband photoacoustic signals detected by a micro-ring after the deconvolution to remove the signal bandwidth associated with the 6 ns laser pulses. The calculated 3 dB receiving bandwidth of the micro-ring resonators reached 173.5 MHz. Based on the measured signal and noise level in the Supplementary Fig. S2(b)), The calculated SNRs at 25.8 MHz, 88.4 MHz and 173.5 MHz are 26.9 dB, 22.4 dB and 12.6 dB, respectively. More detailed descriptions are included in the Supplementary information of Section 2. This broadband receiving capability of the micro-rings, facilitated by the polymer waveguide’s high quality-factor and photo-elastic coefficient, significantly enhances the axial resolution of the acquired PAT images.

We characterized the acoustic sensitivity of the microring device employing a calibrated hydrophone (HNC-1000, Onda) with 1–20 MHz bandwidth and an acoustic transducer with 10 MHz centered frequency (A312S-SU, Olympus), following the procedures described in the reference^1^. The measured peak signal outputs of the hydrophone and the micro-ring resonator were 1.4 mV and 260 mV with 100 times of averaging data acquisition, respectively. As shown in Fig. 5(b), the calculated sensitivity of the micro-ring resonator shows a very high receiving sensitivity of 121.8 μV/Pa at 10 MHz. The noise amplitude spectral density of the micro-ring resonator was measured when the transducer was turned off, and the root mean square (RMS) noise level was 1.5 mV without performing signal averaging. By dividing the noise amplitude spectral density by the sensitivity of the micro-ring, the noise equivalent pressure (NEP) spectral density (NSD) was calculated to be below 6.1 mPa/Hz^1/2^, corresponding to a NEP of 13.5 Pa within 20 MHz bandwidth.

To quantitively assess the imaging performance of the micro-ring resonator array, we quantified the lateral and axial resolution of the reconstructed PAT images as a function of depth and lateral position. To achieve this, we utilized an acoustic point source created with a titanium-coated single mode fiber coupled to a pulsed laser providing 6 ns light pulses. By positioning this acoustic point source at different locations within the field-of-view of the micro-ring array, we acquired the acoustic signals from all 40 microring elements and reconstructed the PAT image of the acoustic point source at each location. This approach allowed us to quantify the lateral and axial resolution of micro-ring array-based PAT as a function of depth and position.

In the combined image shown in Fig. 5(c), we can see the overall spot size of the acoustic point source as a function of axial and lateral positions in the reconstructed PAT image. The image reveals a 3 by 3 grid with the center ones aligned with the center of the micro-ring array, and depths ranging from 1.1 mm to 4.6 mm. Figure 5(d) (top) presents the intensity profiles along both lateral and axial directions of the imaged acoustic point source placed at the depth of 1.1 mm from the center of the micro-ring array. Based on the measurements of the full width at half maximum (FWHM) of the intensity profiles, the lateral and axial resolutions are 40 μm and 38 μm, respectively. These measurements closely align with the theoretical resolution values calculated by the following equations ^30^:

(2) $$R_{axial}=\frac{0.5v_{s}}{f_{c}},$$

(3) $$R_{\text{lateral}}=\frac{v_{s}d}{f_{c}a},$$

where $v_{s}$ is the speed of sound of 1500 m/s in the medium, and $f_{c}$ is the center frequency of micro-rings, which is 20 MHz according to Supplementary Section 2, and a and d are the aperture size of the micro-ring array and the depth, respectively^30^.

Figure 5(d) (bottom) illustrates both the measured and theoretical values of the lateral and axial resolutions at different depths along the center of the micro-ring array (i.e., the three spots along the center column in Fig. 5(c)). The quantified lateral and axial resolutions at the other locations (i.e., the spots at the two side columns in Fig. 5(c)) are shown in Supplementary information of Section 2. The measured axial resolutions at the three different depths are all around 38 μm, and are consistent with the theoretical values. The measured lateral resolutions slightly degrade with the depth, also consistent with the theoretical values. These measurements demonstrate that, by leveraging the broad receiving bandwidth, the micro-ring array facilitates PAT imaging with high resolution along both axial and lateral directions. Since the lateral resolution is also a function of the *f*-number (i.e., the ratio between the imaging depth and the aperture of the array), the number of the micro-ring elements in an array is important to achieve a high lateral resolution.

**Fig. 6 |:**
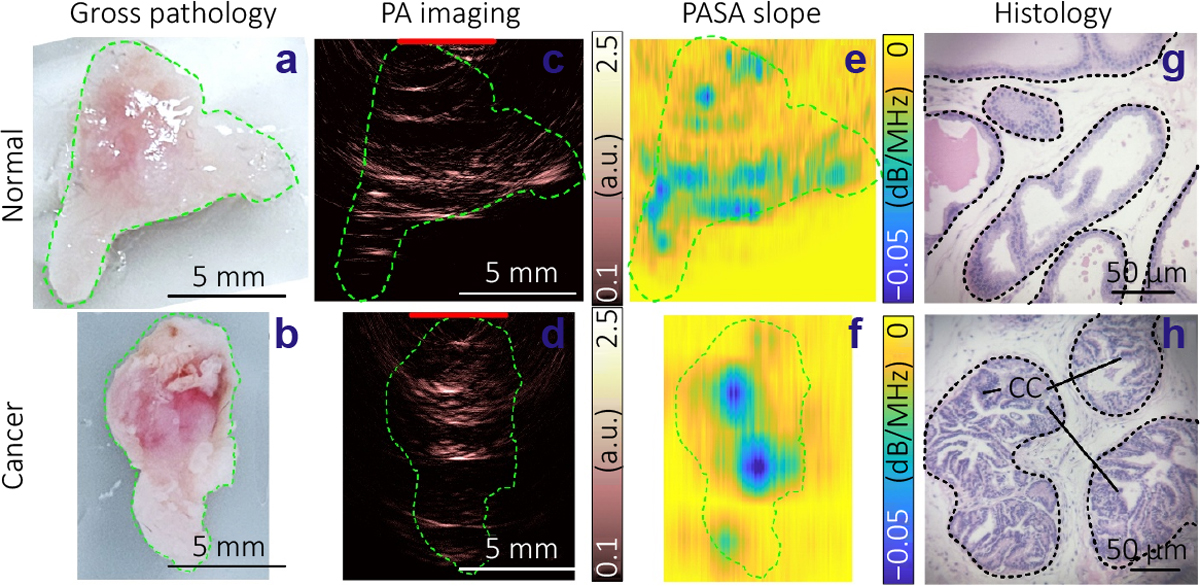
Differentiating normal and cancerous mouse prostate with micro-ring array-based PAT and PASA. (a, b) Gross pathology of normal and cancerous mouse prostates. (c, d) PAT images of the samples in (a, b) at 800 nm. The red lines mark the position of the micro-ring resonator arrays. (e, f) PASA slopes images derived from PAT images. An average slope value was derived from each image for statistics. (g, h) Histology of the samples. The glandular architecture are marked by the dashed contours. The light-color areas between the contours are supporting tissue. CC: cancer cells.

Photoacoustic imaging is regarded as a functional imaging modality, because objects with light absorption contrast excited by different excitation light wavelengths can be differentiated. We examined the feasibility of differentiating normal and cancerous mouse prostates by the micro-ring array-based PAT. By using an optical fiber bundle to deliver pulsed laser light at 800 nm, 1220 nm, and 1370 nm, targeting the hemoglobin, lipid, and collagen contents, respectively, within the entire prostate tissue. Lipid and collagen are rich in the supporting glandular microarchitecture in the prostate, which is highly relevant to the progression of prostate cancer^31^. Figure 6(a, b) shows the gross photos of two mouse prostates. Figure 6(c, d) shows the PAT images, which are produced by envelope detection of the radiofrequency beam-formed PA signals at 800 nm. The envelope detection was achieved by Hilbert transformation. The contours of the samples were delineated in PAT images, which are similar to the gross photos in Fig. 6(a, b).

**Fig. 7 |:**
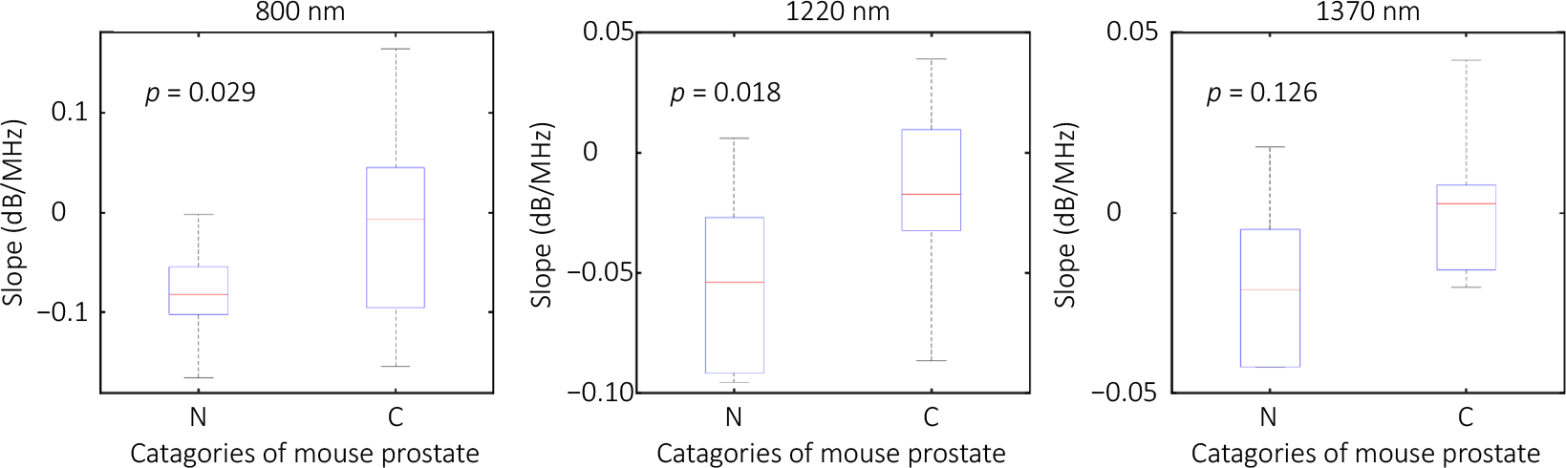
Statistics of PASA slopes derived from 12 normal and 12 cancerous mouse prostates. At each laser wavelength (i.e., 800 nm, 1220 nm, and 1370 nm), a *t*-test is conducted with the null hypothesis that the measurements cannot differentiate the two groups.

The photoacoustic spectral analysis (PASA) method can reveal the size distribution of the objects within the tissue by a Fourier transform of the photoacoustic signal. PASA was applied to the A-lines (i.e., vertical lines) of the radiofrequency beam-formed photoacoustic signals, following the method described in our previous publication^32^. In brief, the frequency domain power spectra of the photoacoustic signals are fitted to linear models. The slope of each linear model reflecting the ratio between high-frequency and low-frequency components in the photoacoustic signals is quantified. In this study, we analyzed signals within the frequency range of 0.5–40 MHz, focusing on the first frequency hump shown in Fig. 5(a). Using a sliding window of 1.8 mm, which is approximately 50 wavelengths of a 40 MHz acoustic wave, we moved in 0.036 mm steps along the signals. For each window, we calculated and calibrated the power spectra of the beamformed PA signals based on the average frequency response of the micro-ring elements. We then fit the power spectra from 0.5 to 40 MHz to linear models. The slopes of these linear models were quantified to represent the dimensions of the microarchitecture in the tissue samples, as illustrated in the histology images in Fig. 6(g) and 6(h). Figure 6(e) and 6(f) display the distribution of the PASA slopes derived from PAT images at 800 nm. These slopes were averaged within the contours of the samples to derive a quantitative measurement for each sample. Figure 7 shows the average PASA slopes derived from 12 normal and 12 cancerous mouse prostate samples. The PASA slopes at 800 nm and 1220 nm show significant differences between the normal and the cancerous groups. The increase in slope value in 26.3–31.8 MHz in the cancerous mouse prostate at 800 nm may be due to the proliferation of the microvasculature^33^ with dimensions covered by this acoustic frequency range. The difference at 1220 nm is believed to be due to the change in gland architecture in cancerous prostate tissues. As we can see in Fig. 6(h), the gland architecture enlarges in cancerous prostates (i.e., the dark purple clusters of cancer cells) and invades the supporting architecture, leading to the decreased dimensions of the supporting architecture^34^. These architectures, with dimensions of tens of microns, are covered by the acoustic frequencies in the 18.5–30.1MHz range. In Fig. 7, the PASA slopes of the two groups at 1370 nm also show an observable difference comparable to that at 1220 nm. This agrees with the fact that both lipid and collagen exist in the supporting tissue architecture. The less significant observation at 1370 may be due to the lower illumination energy and the resulting low signal-to-noise ratio.

## 4 Conclusions

In this study, we demonstrated a polymer-based micro-ring resonator array with 40 controllable elements, fabricated using nanoimprint lithography (NIL). We achieved distinguishable and sequential resonances of the micro-rings over a narrow free spectral range of 2.19 nm, realizing a high-quality factor of 2.34×10^5^. The array device exhibited an acoustic bandwidth of 173.5 MHz, an acoustic sensitivity of 121.8 μV/Pa at 10 MHz, and NEP of 13.5 Pa. Our automated spectrum tracking and acquisition algorithm effectively mitigated spectrum shifts caused by temperature and pressure changes. PAT using the array device achieved lateral and axial resolutions of 40 μm and 38 μm, respectively. The micro-ring array device successfully acquired images of *ex vivo* mouse prostates, which have complex biological structures, without the need to translate the target tissue or the array. Furthermore, by utilizing the PASA method, PAT powered by the high-frequency micro-ring resonator array showed the capability to differentiate cancerous prostates from normal ones. The method for fabricating and controlling wavelength division micro-ring arrays developed in this work holds a great potential for PAT based cancer detection and characterization, as well as in state-of-the-art optical communications and integrated photonic systems.

## 5 Discussions

In our fabrication process, the micro-ring array has a residual layer thickness of 58 nm, which decreases the quality factor of resonances within the micro-ring resonator. We also investigated the quality factor as a function of residual layer thickness. As detailed in the Supplementary Section 4, we found that when the residual layer is less than 10 nm with minimal scattering losses, the quality factor of the micro-rings can reach up to 1.48×10^6^. Therefore, by employing advanced NIL fabrication processes to reduce the residual layer as well as scattering and radiation losses, it is possible to improve the quality factor beyond 2.34×10^5^. Our future work will focus on enhancing the fabrication process to increase the quality factor further, thereby allowing for a larger number of micro-ring resonators in the array.

Due to factors such as system noise and imperfect point source, the measured spatial resolutions in this study are slightly larger than the theoretical values. With 40 elements and a pitch of 100 μm, the aperture of the array is only 4 mm. The limited aperture resulted in a lower lateral resolution and reconstruction artifacts in deeper regions. Our immediate future work will be increasing the element number in the micro-ring array by fine-tuning the design of micro-ring array structures and using the optimized fabrication procedures^22,35^.

For the multi-position acquisitions used to extend the effective FOV or to generate multiple point sources, the combined images (e.g., Fig. 4(e) and Fig. 5(c)) were generated by mechanically repositioning the imaging target with known translation distances. Because the point-source or sample locations were predefined, the mosaicking uncertainty and repeatability are primarily limited by the positioning accuracy of the translation stage, which is 10 μm and below the spatial resolution of the microring imaging system.

Potential strategy involves changing the core material to improve both sensitivity and the total number of usable resonances. In addition, employing higher-refractive index core materials can increase the *Q*-factor and/or reduce the size of the microrings, thereby allowing more densely spaced resonances and supporting a larger array size. At present, we use polystyrene because it cleaves cleanly for edge coupling between the bus waveguide and optical fibers, but this restricts the choice of materials. The use of grating couplers would relax this constraint, allowing broader material selection and facilitating larger arrays.

Even though we reliably obtain spectra from up to 40 microrings, the array still exhibits variations in resonance shape, partial spectral overlap, and occasional non-sequential resonance ordering, all of which complicate device control. These limitations can be mitigated by optimizing the coupling efficiency between waveguides. For example, non-circular shape microrings^2,35,36^ can provide more uniform coupling across rings of different sizes and positions, improving both resonance uniformity and *Q*-factors, which is essential for improving sequential controllability and non-sequential order.

Although our work demonstrates a fully controllable microring resonator array, the current system still relies on sequential wavelength scanning of the tunable laser, resulting in an acquisition time of approximately 7 minutes. The scanning speed can be substantially improved through several strategies. One approach is to employ a comb laser source, which provides multiple discrete wavelengths simultaneously and enables parallel interrogation of all microrings, as previously demonstrated by Pan J et al.^1^ using a 15-element array. This strategy could eliminate mechanical tuning delays and ultimately enable real-time, more sensor elements, 2D array configuration, and motion-robust in-vivo photoacoustic imaging.

We used mouse prostates to showcase the broad bandwidth and high sensitivity of the microring resonator array. Using 532 nm illumination, the PAT system successfully resolved the distribution of the hemoglobin content within the mouse prostate as seen in the gross pathology. Multi-wavelength PASA targeting hemoglobin, lipid and collagen shows that the device possesses sufficient bandwidth to characterize the tissue architecture in the prostates to differentiate the normal and cancerous groups. Due to the limited sample size for initial validation study, we performed *t*-test, which is designed for small to medium sample sizes. In our future work, we will further miniaturize the microring resonator array for *in vivo* studies, and included a large sample size and more comprehensive statistics.

The small element size and all optical design of the microring resonator array holds great promise in future developments of miniaturized and/or disposable PAT imaging devices.

## Supplementary Material

Supple

Video

Supplementary information

Supplementary information related to this article can be found online. https://doi.org/10.29026/oea.2026.250215

## References

[1] Pan JS, Li Q, Feng YM Parallel interrogation of the chalcogenide-based micro-ring sensor array for photoacoustic tomography. Nat Commun 14, 3250 (2023). 37277353 10.1038/s41467-023-39075-3 PMC10241812

[2] Yuan Y, Peng YW, Sorin WV A 5 × 200 Gbps microring modulator silicon chip empowered by two-segment Z-shape junctions. Nat Commun 15, 918 (2024). 38297012 10.1038/s41467-024-45301-3 PMC10831040

[3] Pintus P, Dumont M, Shah V Integrated non-reciprocal magneto-optics with ultra-high endurance for photonic in-memory computing. Nat Photonics 19, 54–62 (2025).

[4] Luo LW, Ophir N, Chen CP WDM-compatible mode-division multiplexing on a silicon chip. Nat Commun 5, 3069 (2014). 24423882 10.1038/ncomms4069

[5] Jia H, Yang SL, Zhou T WDM-compatible multimode optical switching system-on-chip. Nanophotonics 8, 889–898 (2019).

[6] Ohno S, Tang R, Toprasertpong K Si microring resonator crossbar array for on-chip inference and training of the optical neural network. ACS Photonics 9, 2614–2622 (2022).

[7] Westerveld WJ, Mahmud-Ul-Hasan M, Shnaiderman R Sensitive, small, broadband and scalable optomechanical ultrasound sensor in silicon photonics. Nat Photonics 15, 341–345 (2021).

[8] Choi S, Kim J, Jeon H Advancements in photoacoustic detection techniques for biomedical imaging. npj Acoust 1, 1 (2025).

[9] Huang LY, Yang CJ, Liang L Integrated light sources based on micro-ring resonators for chip-based LiDAR. Laser Photonics Rev 19, 2400343 (2025).

[10] Mahmudlu H, Johanning R, van Rees A Fully on-chip photonic turnkey quantum source for entangled qubit/qudit state generation. Nat Photonics 17, 518–524 (2023).

[11] Chen R, Fang ZR, Perez C Non-volatile electrically programmable integrated photonics with a 5-bit operation. Nat Commun 14, 3465 (2023). 37308496 10.1038/s41467-023-39180-3 PMC10261021

[12] Guo LJ, Chao CY. Biochemical sensors with micro-resonators. US patent US 2006/0170931 A1. (2006).

[13] Tu X, Chen SL, Song CL Ultrahigh *Q* polymer microring resonators for biosensing applications. IEEE Photonics J 11, 4200110 (2019).

[14] Zhang C, Ling T, Chen SL Ultrabroad bandwidth and highly sensitive optical ultrasonic detector for photoacoustic imaging. ACS Photonics 1, 1093–1098 (2014).

[15] Liang S, Park K, Lin W Photoacoustic sensing and transmission for biological and industrial applications. Device 3, 100831 (2025).

[16] Huang SW, Chen SL, Ling T Low-noise wideband ultrasound detection using polymer microring resonators. Appl Phys Lett 92, 193509 (2008). 19479044 10.1063/1.2929379 PMC2682739

[17] Chen SL, Guo LJ, Wang XD. All-optical photoacoustic microscopy. Photoacoustics 3, 143–150 (2015). 31467845 10.1016/j.pacs.2015.11.001 PMC6713062

[18] Xie ZX, Chen SL, Ling T Pure optical photoacoustic microscopy. Opt Express 19, 9027–9034 (2011). 21643156 10.1364/OE.19.009027 PMC3324262

[19] Li H, Dong BQ, Zhang X Disposable ultrasound-sensing chronic cranial window by soft nanoimprinting lithography. Nat Commun 10, 4277 (2019). 31537800 10.1038/s41467-019-12178-6 PMC6753120

[20] Nagli M, Moisseev R, Suleymanov N Silicon photonic acoustic detector (SPADE) using a silicon nitride microring resonator. Photoacoustics 32, 100527 (2023). 37645254 10.1016/j.pacs.2023.100527 PMC10461202

[21] Chao CY, Guo LJ. Reduction of surface scattering loss in polymer microrings using thermal-reflow technique. IEEE Photonics Technol Lett 16, 1498–1500 (2004).

[22] Lin WK, Liu S, Lee S High Q-factor polymer microring resonators realized by versatile damascene soft nanoimprinting lithography. Adv Funct Mater 34, 2312229 (2024). 39022395 10.1002/adfm.202312229 PMC11251712

[23] Wei HM, Krishnaswamy S. Polymer micro-ring resonator integrated with a fiber ring laser for ultrasound detection. Opt Lett 42, 2655–2658 (2017). 28957308 10.1364/OL.42.002655

[24] Gonzalez-Hernandez D, Varapnickas S, Bertoncini A Microoptics 3D printed via multi-photon laser lithography. Adv Opt Mater 11, 2201701 (2023).

[25] Otuka AJG, Tomazio NB, Paula KT Two-photon polymerization: functionalized microstructures, micro-resonators, and bio-scaffolds. Polymers 13, 1994 (2021). 34207089 10.3390/polym13121994 PMC8234590

[26] Guo LJ. Nanoimprint lithography: methods and material requirements. Adv Mater 19, 495–513 (2007).

[27] Lin W-K, Guo LJ. 30 years of nanoimprint: development, momentum and prospects. Opto-Electron Technol 1, 250001 (2025).

[28] Kwon B, Kim JH. Importance of molds for nanoimprint lithography: hard, soft, and hybrid molds. J Nanosci 2016, 6571297 (2016).

[29] Chou SY, Krauss PR, Renstrom PJ. Imprint of sub-25 nm vias and trenches in polymers. Appl Phys Lett 67, 3114–3116 (1995).

[30] Giurgiutiu V. Chapter 13 - In situ phased arrays with piezoelectric wafer active sensors. In Giurgiutiu VG. Structural Health Monitoring with Piezoelectric Wafer Active Sensors (Second Edition) 707–805 (Elsevier, Amsterdam, 2014).

[31] Ni LY, Lin WK, Kasputis A Assessment of prostate cancer progression using a translational needle photoacoustic sensing probe: preliminary study with intact human prostates ex-vivo. Photoacoustics 28, 100418 (2022). 36386297 10.1016/j.pacs.2022.100418 PMC9650056

[32] Jo J, Siddiqui J, Zhu YH Photoacoustic spectral analysis at ultraviolet wavelengths for characterizing the Gleason grades of prostate cancer. Opt Lett 45, 6042–6045 (2020). 33137064 10.1364/OL.409249 PMC7687867

[33] Børretzen A, Reisæter LAR, Ringheim A Microvascular proliferation is associated with high tumour blood flow by mpMRI and disease progression in primary prostate cancer. Sci Rep 13, 17949 (2023). 37863961 10.1038/s41598-023-45158-4 PMC10589248

[34] Pu Y, Wang WB, Tang GC Changes of collagen and nicotinamide adenine dinucleotide in human cancerous and normal prostate tissues studied using native fluorescence spectroscopy with selective excitation wavelength. J Biomed Opt 15, 047008 (2010). 20799839 10.1117/1.3463479

[35] Zhang LC, Chen JM, Ma WC Low-loss, ultracompact n-adjustable waveguide bends for photonic integrated circuits. Opt Express 31, 2792–2806 (2023). 36785285 10.1364/OE.475398

[36] Zhang YJ, Zhong KY, Zhou XT Broadband high-Q multimode silicon concentric racetrack resonators for widely tunable Raman lasers. Nat Commun 13, 3534 (2022). 35725566 10.1038/s41467-022-31244-0 PMC9209424

