# Supplementary material for: Imprinted high-Q polymer micro-ring resonator array for high-resolution photoacoustic tomography
Source: Opto Electron Adv. Author manuscript; Available in PMC 2026 Jun 7. (PMC13242253; doi:10.29026/oea.2026.250215)
Supplement: Supple [file NIHMS2170367-supplement-Supple.pdf]

Opto-Electronic Advances

Supplementary information

June 2026, Vol. 9, No. 6

Open Access

Imprinted high-*Q* polymer micro-ring resonator array for high-resolution photoacoustic tomography

Hyeonwoo Kim[1†], Wei-Kuan Lin[1†], Linyu Ni[2†], Mohammad Ali[1], Xueding Wang[2,3]*, Guan Xu[2,4]* and L. Jay Guo[1]*

[1]Department of Electrical Engineering and Computer Sciences, University of Michigan, 1301 Beal Avenue, Ann Arbor, MI, USA; [2]Department of Biomedical Engineering, University of Michigan, 2200 Bonisteel Blvd, Ann Arbor, MI, USA; [3]Department of Radiology, University of Michigan, 1301 Catherine St, Ann Arbor, MI, USA; [4]Department of Ophthalmology and Visual Sciences, University of Michigan, 1000 Wall St, Ann Arbor, MI, USA.

†These authors contributed equally to this work.

*Correspondence: XD Wang, E-mail: xdwang@umich.edu; G Xu, E-mail: guanx@med.umich.edu; LJ Guo, E-mail: guo@umich.edu

This file includes:

Section 1: Fabrication procedures
Section 2: Characterization process of acoustic responses
Section 3: Details for control micro rings
Section 4: High quality factor
Section 5: Packaging process and insertion loss
Section 6: Sensitivity and NEP uniformity across the 40 sensors
Section 7: Si mold and RIE optimization
Section 8: Scanning stability of the thermal induced short-term fluctuations on microrings

Supplementary information for this paper is available at https://doi.org/10.29026/oea.2026.250215

Section 1: Fabrication procedures

We utilized JEOL JBX-6300FS electron-beam lithography to fabricate a high-quality mold. The fabrication process of the mold begins with spin coating a 500 nm layer of positive electron beam lithography (EBL) resist (ZEP 520A from ZEON corp.) onto a silicon wafer, as shown in Fig. S1(a). This resist is then exposed to an electron beam at 100 kV and a dose of 250 nA/cm^2. Following exposure, the resist undergoes development with ZED-N50 (also from ZEON corp.), a process completed in 120 seconds, after which it is rinsed with isopropyl alcohol and dried with nitrogen gas. The next step involves reactive ion etching (RIE) using the Lam Research LAM 9400 system, which etches a 1.4 μm deep waveguide pattern utilizing 100 sccm of both HBr and He gases at 30 mTorr and 600 W of TCP power. Post-etching, the mold is treated with buffered hydrofluoric acid for 30 seconds to etch the oxide layer, followed by a 30-minute nanostrip treatment to eliminate residual e-beam resist. Finally, the mold is treated with an anti-sticking agent, specifically Perfluorooctyl trichlorosilane (ThermoFisher, Perfluorooctyl trichlorosilane, 97%), incubated at 150 °C for one hour.

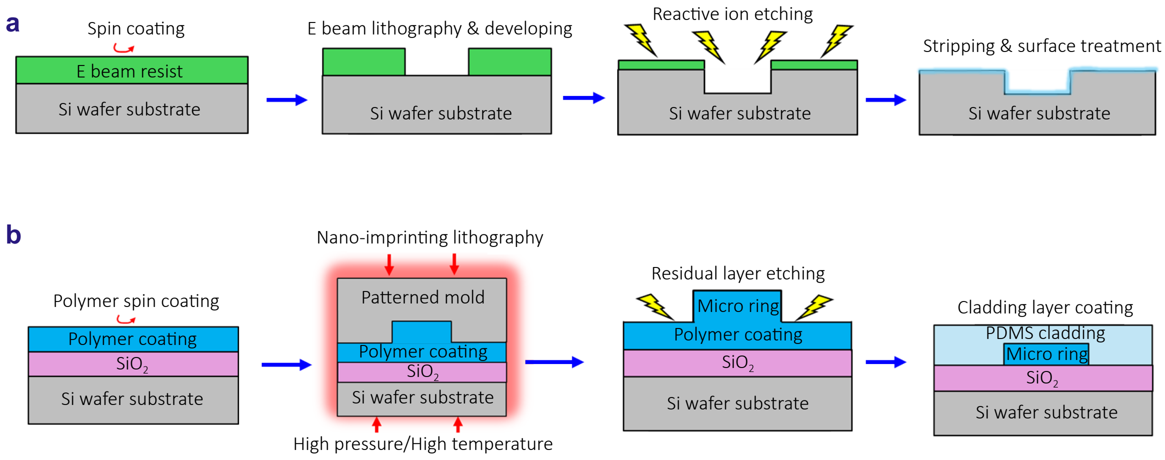

Fig. S1 | (a) Schematic diagrams for fabrication steps of silicon mold patterned with micro ring arrays using EBL and (b) micro ring array on 2 μm SiO_2 layer on silicon wafer using NIL.

For the imprinting process, this silicon mold is used to stamp a micro ring array onto a silicon substrate employing a 3 wt% polystyrene solution (MW 50,000, Polysciences, diluted in toluene). This material was chosen for its high refractive index of 1.577 at a wavelength of 780 nm, excellent transparency, and high photo-elastic coefficient, making it ideal for high-quality factor resonance and acoustic-sensitive applications. As illustrated in Fig. S1(b), the polystyrene solution is spin-coated onto a silicon wafer already layered with 2 μm of SiO_2, resulting in a 250 nm polymer film thickness. The silicon mold then undergoes thermal imprint lithography with the Nanonex NX-2000 system at 160 °C and 350 PSI for 90 seconds to form the patterned polystyrene waveguides and rings. Following the imprinting, reactive ion etching with oxygen reduces the residual layer to less than 100 nm, thereby minimizing radiation loss and preserving the high-quality factors of the micro rings. Upon completing the fabrication process, the micro ring array chips are coated with a 2 μm layer of PDMS precursor (Dow Corning, Sylgard 184, 10:1 mixing ratio), which acts as the cladding layer for the waveguides.

Section 2: Characterization process of acoustic responses

To measure the acoustic signals, Fig. S2(a) illustrates the experimental setup. A continuous-wave 780 nm tunable laser (New Focus, TLB-6312) was connected to a polarization-maintaining fiber (PMF), which was coupled to the tapered waveguide of the micro ring array device. The output power propagating through the waveguides was collected by a multi-mode fiber (MMF), a photo detector (Newport, 1801-FC-AC), and a digital oscilloscope (Tektronix, MSO44). The wavelength of the tunable laser was positioned on the slope of the resonant wavelength of the micro ring array, corresponding to the median quality factor of all the micro rings. To generate the broadband ultrasound signal, a 100 nm thick gold film on a glass substrate was placed on the micro ring array device with water. A pulsed laser (Amplitude, Surlite I-20) with a wavelength of 532 nm was illuminated on the gold film that absorbs the laser pulses, and the absorbed energy generated an ultrasound signal. Since the laser pulse duration, which is typically 6 ns, is much longer than the acoustic transient time of the gold layer, the generated ultrasound signal is similar to the incident pulsed laser in time domain. This implies that the −3 dB acoustic

frequency bandwidth of pulse laser is 88.4 MHz. As we can see in Fig. S2(b), the calibrated amplitude spectral density of micro ring resonator can be obtained by dividing the measured amplitude spectral density of micro ring (red solid line) by the normalized amplitude spectrum density of pulse laser (blue solid line). As a result, the measured −3 dB bandwidth of micro ring resonator before calibration is 122 MHz, and the calculated −3 dB bandwidth of micro ring resonator after calibration is 173.5 MHz.

We characterized the sensitivity of the micro ring device, employing a calibrated hydrophone (Onda corp., HNC-1000) with 1-20 MHz bandwidth and an acoustic transducer (Olympus, A312S-SU). Using a calibrated hydrophone, we determine the acoustic pressure generated by the transducer. The sensitivity of the microring resonator is then calculated by dividing the measured output of the microring by the corresponding acoustic pressure. The sensitivity of the calibrated hydrophone is 0.56 μV/Pa without pre-amplification, and the applied voltage on the transducer is 2 V using a function generator (HP 3314A Function Generator), corresponding to a pressure of 2.5 kPa. The peak signal outputs of the hydrophone and the micro ring resonator were 1.4 mV and 260 mV with 100 times of averaging data acquisition, respectively.

Figure S2(c) presents the experimental schematic diagram used to investigate the lateral and axial resolution of the micro-ring array device. An optical fiber with a mode field diameter of 10.4 μm at a wavelength of 1550 nm (Thorlabs, SMF28 Ultra), coated with a 150 nm thick layer of titanium film, was used to generate a point photoacoustic source by coupling it with a pulsed laser at a wavelength of 532 nm. The tip of the fiber was moved and positioned at a combination of three lateral and three axial locations. Figure S2(d) and S2(e) show the lateral and axial resolution measured at the left and right positions as functions of depth, respectively. Since these spots were not aligned with the center of the micro-ring resonator array, the field of view of each micro-ring was limited in capturing the photoacoustic signals from these locations, leading to degraded lateral resolution. However, this underscores the importance of having a large number of micro-ring resonators to enlarge the array's field of view. Figure S2(f) illustrates the photoacoustic signal from the fiber tip and its amplitude spectral density. The central frequency f_c of the signal is 20 MHz.

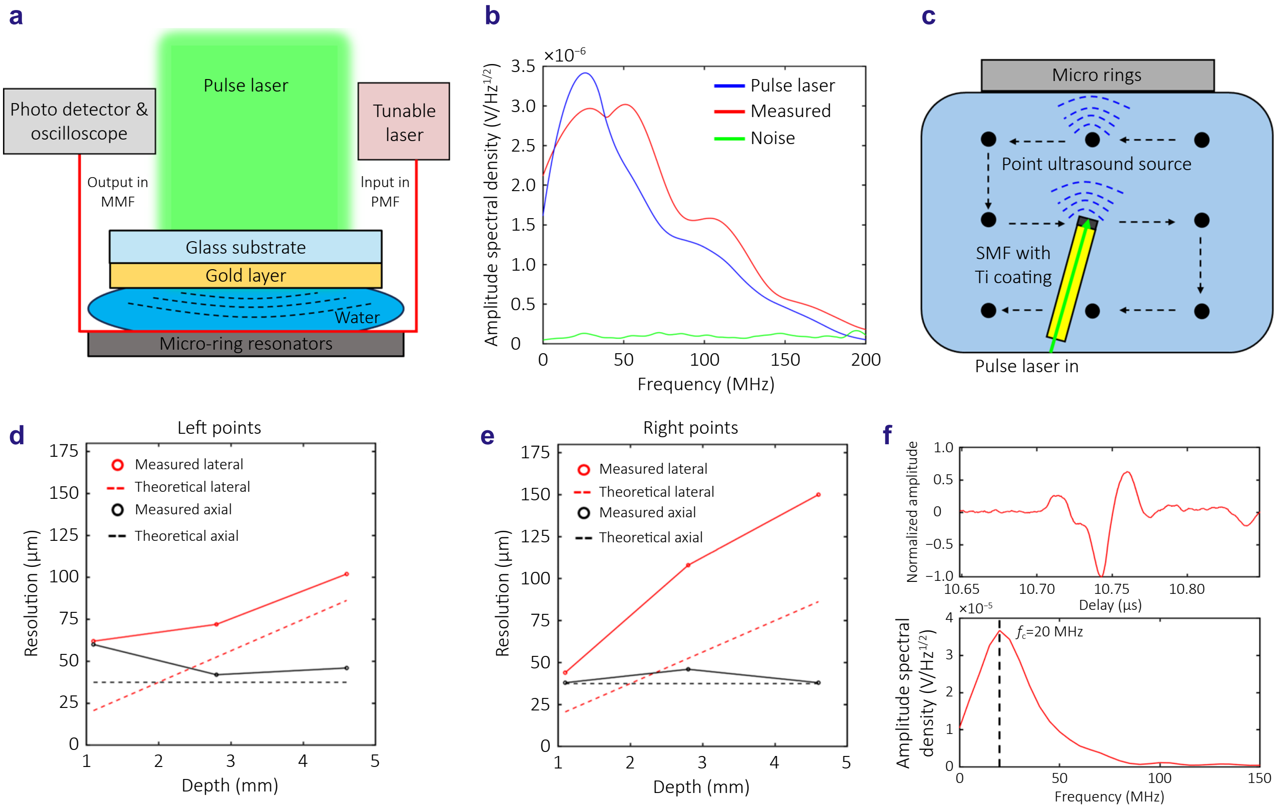

Fig. S2 | (a) Experimental schematic diagram for frequency response measurement. (b) The measured amplitude spectral density of the micro ring resonator (red solid line), pulse laser response (blue solid line) and noise (green solid line). (c) Experimental configuration for measuring 3 by 3 resolution distribution and (d) resolution of left points and (e) right points. (f) The photoacoustic signal from the tip of optical fiber with pulse laser and the frequency domain response of the acoustic signal.

Section 3: Details for control micro rings

Figure S3(a) and S3(b) illustrate how the spectrum changes as a function of applied pressures and changing of temperatures. Surrounding conditions affect the refractive index of the micro ring, causing the overall spectrum to shift to longer or shorter wavelengths. This shift complicates positioning the tunable laser's wavelength to the resonant wavelengths of each micro ring resonator. However, we observed that this temperature and pressure effect primarily results in wavelength shifts without affecting the sequence order of the micro rings, provided that the scanning time for all rings is sufficiently fast. To calculate the amount of shift, we utilized the loss function value between the reference and shifted spectrum using the following equation:

$$MSE\,(\Delta\lambda) = \sum_{\lambda=\lambda_1}^{\lambda_2} \left(Y'(\lambda - \Delta\lambda) - Y(\lambda)\right)^2 , \quad \text{(S1)}$$

$Y'(\lambda)$ represents the shifted spectrum, while $Y(\lambda)$ is the reference spectrum; λ is the wavelength ranging from λ_1 (the shortest) to λ_2 (the longest). The spectrum shift value ($\Delta\lambda$) can be determined by finding the λ_{shift} that minimizes the mean squared error (MSE) between Y' and Y. Figure S3(c) shows how the spectrum shifts over a 15-minute period when water is placed on the micro ring resonator array at room temperature. After 15 minutes, changes in water temperature and volume led to variations in temperature and pressure on the device, resulting in a 4.7 pm spectral shift due to changes in the refractive index. As illustrated in Fig. S3(d), the calculated wavelength shifts using the minimum MSE method closely match the observed shifts in Fig. S3(c). This approach enables accurate calculation and tracking of spectral shifts, ensuring stable operation of the micro ring array for photoacoustic imaging in a water environment.

By utilizing the spectrum shift values and sequence information of the micro rings, the micro ring resonator array can be precisely controlled. We achieve rapid acquisition of ultrasound signals by automatically positioning the tunable laser's wave-

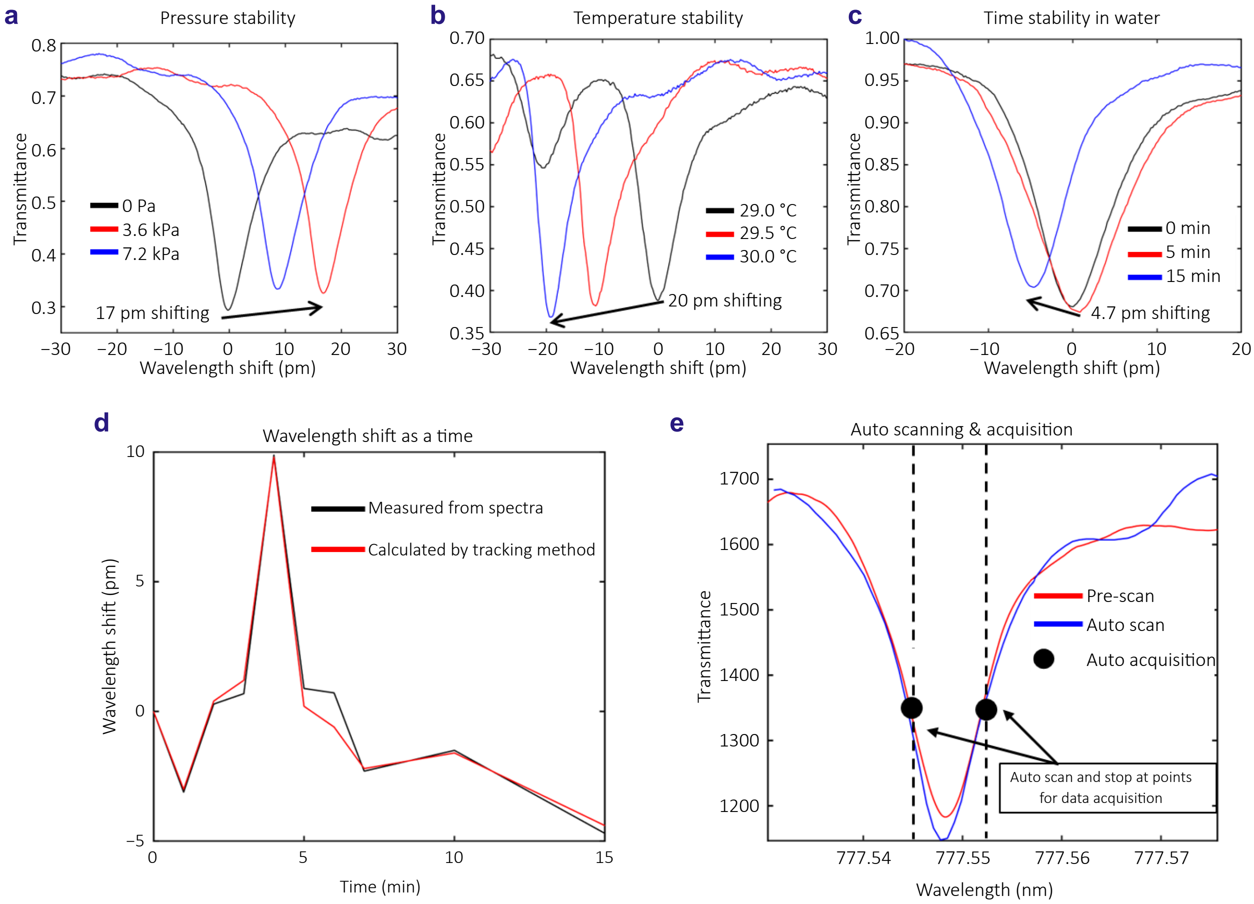

Fig. S3 | Wavelength shift of micro ring resonator array as a function of (a) applied pressure on micro ring, (b) temperature and (c) time stability in water. (d) Wavelength shift comparison between measured from (c) and calculated using the proposed tracking method. (e) Auto scanning and data acquisition method.

length. This process involves a two-step procedure: first step, fast scanning using motor control brings the laser wavelength close to the resonance peaks, and then fine-tuning with piezoelectric control precisely aligns the wavelength to the target resonances, as illustrated in Fig. S3(e). After each measurement, the laser automatically adjusts to the next resonance, enabling us to acquire signals from all 40 elements in the array within 7 minutes.

The total duration of approximately 7 minutes was primarily attributed to step 1 and step 2 of the measurement process. Specifically, step 1, which involved tracking and positioning the laser at the resonant wavelengths of the microrings, required 172.24 s, while step 2, corresponding to data acquisition from the designated wavelengths, took 206.86 s. Furthermore, the computation of the loss-function value is highly efficient, requiring less than 0.5 s for all 40 microrings. The time budget of the system can be divided into wavelength sweeping and positioning at the resonant wavelengths, signal averaging, and data readout/transfer. Wavelength sweeping and positioning required 217.1 s per frame for the interrogation of 40 microring resonators. Signal averaging performed by the oscilloscope took 80 s per frame, while data readout and transfer from the oscilloscope to the PC required an additional 82 s. During imaging, the acquired photoacoustic signals were reconstructed using DAS algorithm, which typically required 1.17 s for a FOV of 4 mm × 100 mm with a pixel size of 5 μm.

Regarding step 1, the overall efficiency can be significantly improved by using a comb laser source, which simultaneously delivers multiple wavelengths to interrogate all microring resonators in parallel. For instance, Pan J et al. [S1] demonstrated a photoacoustic imaging system employing a 15-element microring resonator array and a comb laser source, enabling simultaneous signal acquisition from multiple resonators. As a result, they demonstrated a real-time PAT with a frame rate below 1 ms.

In step 2, most of the time consumption originated from data communication between the PC and the oscilloscope as well as signal averaging, accounting for approximately 162 s of waiting time. The total acquisition time can be further reduced by employing a high-speed DAQ card, and minimizing the averaging process with higher-coupling efficiency microring devices by utilizing grating couplers.

The tunable laser used in our experiment operates on the principle of an external-cavity configuration, in which the lasing wavelength is determined by the optical feedback between the gain medium and a wavelength-selective element in the external cavity. In this design, the external cavity mirror (or grating) is rotated to vary the optical path length and the feedback angle, thereby selecting a specific lasing wavelength that satisfies the cavity resonance condition.

The specific model employed in our setup was TLB 6312 (New Focus Velocity series, Newport Corporation). This tunable laser incorporates a microprocessor-controlled DC servo motor for coarse wavelength tuning and a piezoelectric actuator for fine wavelength tuning. The servo motor rapidly adjusts the external cavity mirror angle to achieve broad and continuous wavelength scanning over the 765–781 nm range, while the piezo actuator provides sub-picometer-scale fine tuning of the mirror position.

Section 4: High quality factor

The results of quality factor measurements for micro rings fabricated under different conditions, along with their corresponding spectra, are shown in Fig. S4. For the micro ring silicon mold fabricated with the initial CF_4 + SF_6 recipe, the measured quality factor is 8,000, as indicated in gray bar. This quality factor is mainly limited by scattering loss. Using fabrication methods with HBr flow shows significant improvement with a quality factor of 69,000 as shown in Fig. S4(a) and Fig. S4(b) red line and bar. Since the residual layer thickness was 250 nm, the radiation loss of micro ring affects the quality factor. After further etching the residual layer under 10 nm thickness, the quality factor improved with 390,000. To address scattering loss caused by sidewall roughness, we began with a thin polymer (150 nm) and applied plasma etching followed by thermal reflow. This process enhanced the quality factor to 1,480,000, marking a record-high quality factor for polymer micro ring resonators, as shown in Fig. S4(a, b) blue line and bar.

To investigate this effect, we simulated a microring waveguide with a radius of 60 μm. As shown inFig. S4(c), a residual free waveguide ensures strong optical confinement within the core region. In contrast, a thicker residual layer, combined with the curvature of the microring, allows a portion of the optical mode to leak into the residual region. This increases radiation loss, which leads to a reduction in *Q*-factor. Additional factors such as power confinement due to refractive index contrast, coupling efficiency, and mode number also contribute to the *Q*-factor. Consistent with these mechanisms, Fig. S4(d) shows that the simulated *Q*-factor decreases from approximately 1.68×10^6 for a 10 nm residual layer to 8.9×10^5 for a 100 nm residual layer.

A thinner residual layer is essential for achieving high acoustic sensitivity of the microring resonator, as the sensitivity is proportional to both the photoelastic coefficient of the core material and the optical *Q*-factor of the resonator. The acoustic sensitivity of the resonator follows this equation:

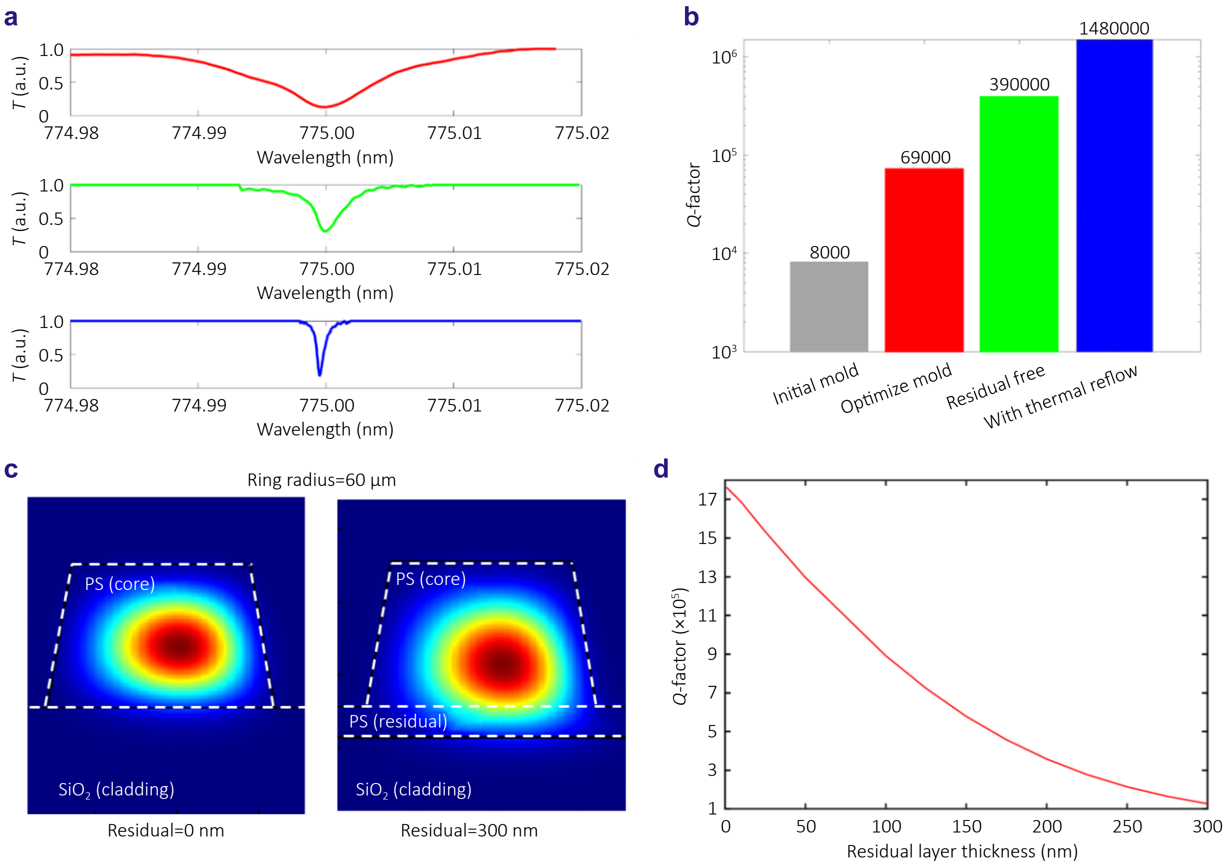

Fig. S4 | (a) Micro ring spectrum with different fabrication recipes. (b) Quality factors. (c) Simulated modes in the microring waveguides, and (d) simulated *Q*-factor as a function of the residual layer thickness.

$$S_p = \frac{\mathrm{d}T}{\mathrm{d}P} \propto \frac{\mathrm{d}n_{\mathrm{eff}}}{\mathrm{d}P} \times Q_{\mathrm{factor}} \ , \tag{S2}$$

where P is the applied acoustic pressure, T is the transmittance from microring and n_{eff} is the effective refractive index of the microring resonator. Lee L et al.[S2] theoretically and experimentally demonstrated that achieving high *Q*-factors is critical for enhancing sensitivity and reducing the noise-equivalent pressure. Based on their findings, minimizing the residual layer thickness plays a key role in achieving high acoustic sensitivity.

Furthermoe, the thickness of the residual layer also influences the acoustic bandwidth. The acoustic cutoff frequency can be approximated as

$$f_t = \frac{v}{2h} \ , \tag{S3}$$

where v is the speed of sound in the surrounding medium and h is the effective acoustic waveguide thickness. A thicker residual layer increases h, thereby reducing the acoustic bandwidth. But since the residual layer is much thinner than that of the waveguide height, the effect is relatively insignificant, unless the goal is to achieve very broadband response.

Section 5: Packaging process and insertion loss

The output power of the tunable laser was 10 mW. After passing through the polarization controller, in-line polarizer, and polarization-maintaining fiber, the optical power coupled into the input waveguide of the microring device was reduced to approximately 5 mW. The transmitted optical power measured from the output waveguide was typically around 300 µW, with a maximum of 600 µW, corresponding to a total fiber-to-fiber insertion loss of approximately 9.2–12.2 dB in the measurement setup.

As we can see in Fig. S5, the device packaging process was designed to ensure mechanical stability and precise optical alignment. First, the microring chip was mounted and bonded on a glass substrate to provide structural support. A polarization-

maintaining fiber (input) and a multimode fiber (output) were then bonded onto glass substrates and carefully aligned to the waveguide facets for optimal coupling efficiency. To prevent positional misalignment caused by deformation of the UV-curable adhesive glue, the fibers were gently pressed against the chip edge to apply frictional forces to resist the deformation of glue. Once the optimal coupling position was achieved, a small amount of optical adhesive was applied on the facets and UV-cured for 0.5 s, followed by a 20 s pause to minimize shrinkage and thermal stress. This curing cycle was carefully repeated ten times to ensure complete polymerization and stable adhesion.

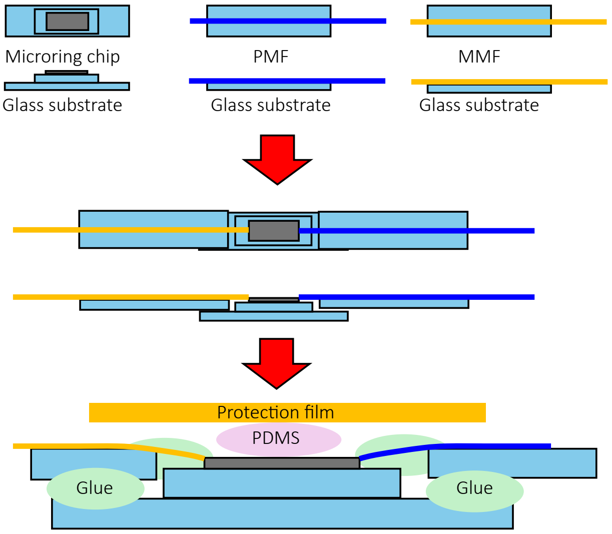

Fig. S5 | Device packaging process for PAT imaging.

After the optical fibers were securely fixed, additional optical adhesive glue was used to bond the glass substrates together. Finally, the packaged device was encapsulated with a thin PDMS layer and covered with a protective film to protect the device during handling and operation.

Section 6: Sensitivity and NEP uniformity across the 40 sensors

The sensitivity of a microring resonator is governed by both the photoelastic coefficient of the core material and the optical *Q*-factor. As shown in Fig. 2(c), the *Q*-factors of the microring array range from 84,000 to 498,000, and the corresponding sensitivities and NEP values follow similar trends. We experimentally determined the peak sensitivities and NEP values by interrogating each microring under a 10 MHz acoustic excitation. Figure S6 summarizes these results, showing that the sensitivities span 72–332 μV/Pa, while the NEP values range from 4.5 to 20.2 Pa across the array.

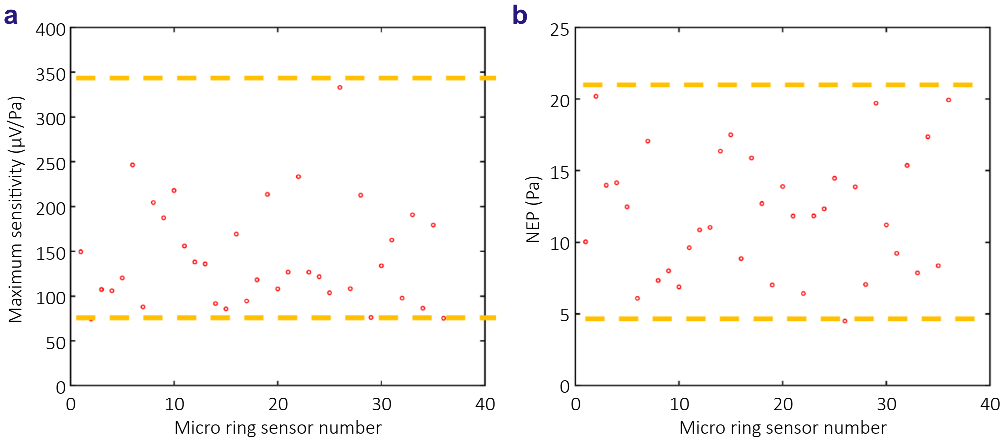

Fig. S6 | (a) Sensitivity and (b) NEP value distribution of 40 microring resonators.

Section 7: Si mold and RIE optimization

Nanoimprint produces a faithful polymer replica of the mold features due to the conformal nature of the NIL process. Therefore, the key to achieve the high precision in the microring size definition is to fabricate high quality Si mold fabrication by e-beam lithography and dry etching, especially the optimization of etching recipe. We found that an initial deep silicon etching process based on an SF_6/C_4F_8 plasma with alternating etching and passivation cycles led to surface roughness of the mold that directly limits the pattern fidelity achievable during NIL.

To improve the mold quality, we adopted an HBr-based Si etching recipe, which is reported to produce smoother and more uniform sidewalls in poly-Si and trench etching applications due to the formation of a Br-rich passivation film. In our experiments, this recipe yielded significantly smoother surfaces and more circular microring profiles, eliminating the polygonal artifacts observed with the SF_6/C_4F_8 process. These improvements are clearly shown in Figure S7.

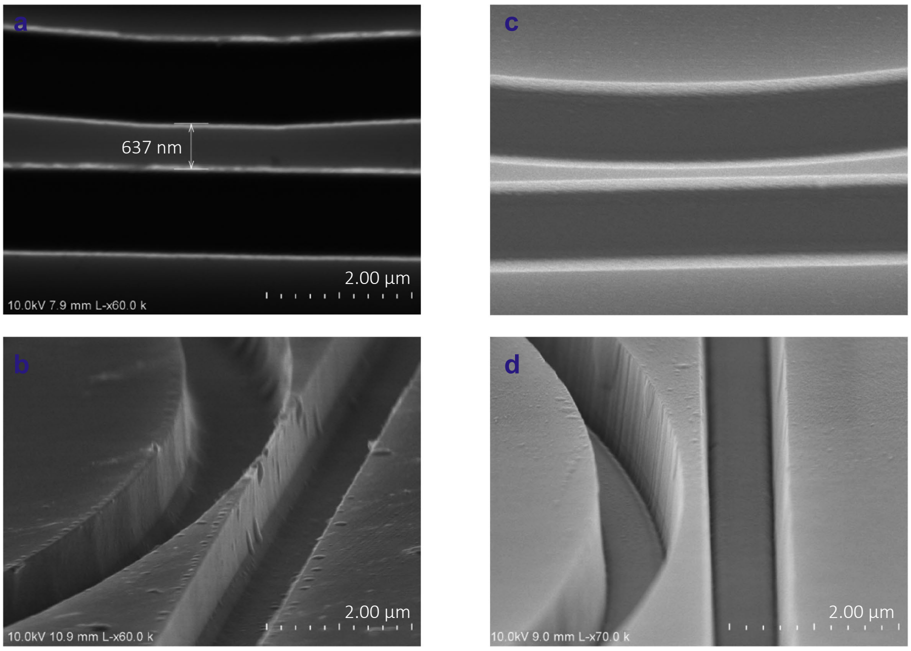

Fig. S7 | Si mold fabricated by SF_6 and C_cF_8 with the top-down view (a) and tilted view (b). Si mold fabricated by HBr gas with the top-down view (c) and tilted view (d).

In addition, during the O_2 reactive ion etching step to reduce the residual layer on imprinted polymer structures, both the RF power and etching duration were precisely tuned to reduce sidewall roughness and maintain accurate pattern transfer from the mold. These optimizations improved the fidelity of the replicated patterns and allowed us to achieve a gradual radius increment of < 2 nm.

Section 8: Scanning stability of the thermal induced short-term fluctuations on microrings

The short-term fluctuations primarily originate from transient thermal effects caused by absorption of the pulsed excitation laser in the Si substrate. The locally generated heat of the Si wafer propagates through the SiO_2 layer on the Si substrate, momentarily changing the refractive index near the microrings and resulting in wavelength shifts. These fluctuations appear as minor distortions in the resonance spectra. Our spectrum tracking algorithm detects and compensates for such fluctuations by calculating the MSE of spectral fitting. For small perturbations, the algorithm successfully tracks the resonant shift with only a slight increase in MSE, ensuring reliable readout across all microrings. However, at higher pulse energies, stronger thermal fluctuations induce larger spectral distortions that may exceed the MSE threshold, leading to potential tracking ambiguity. Representative examples of low- and high-fluctuation cases are shown in Fig. S8(a, b).

As shown in Fig. S8(a), the overall shape of the spectrum under strong fluctuation (blue solid line) closely resembles that of the reference spectrum (red solid line), except for the appearance of sharp spikes at the resonant wavelengths. These distortions increase the MSE in spectral fitting, which may lead to misidentification of the shifted resonances. To mitigate this issue, we optimized the reference spectrum tracking window to a range of 200–250 pm, which effectively prevents false convergence to incorrect resonant wavelengths and stabilizes the resonance tracking process.

To increase the stability of the short-term fluctuations, we minimized the laser absorption in the Si substrate by adjusting

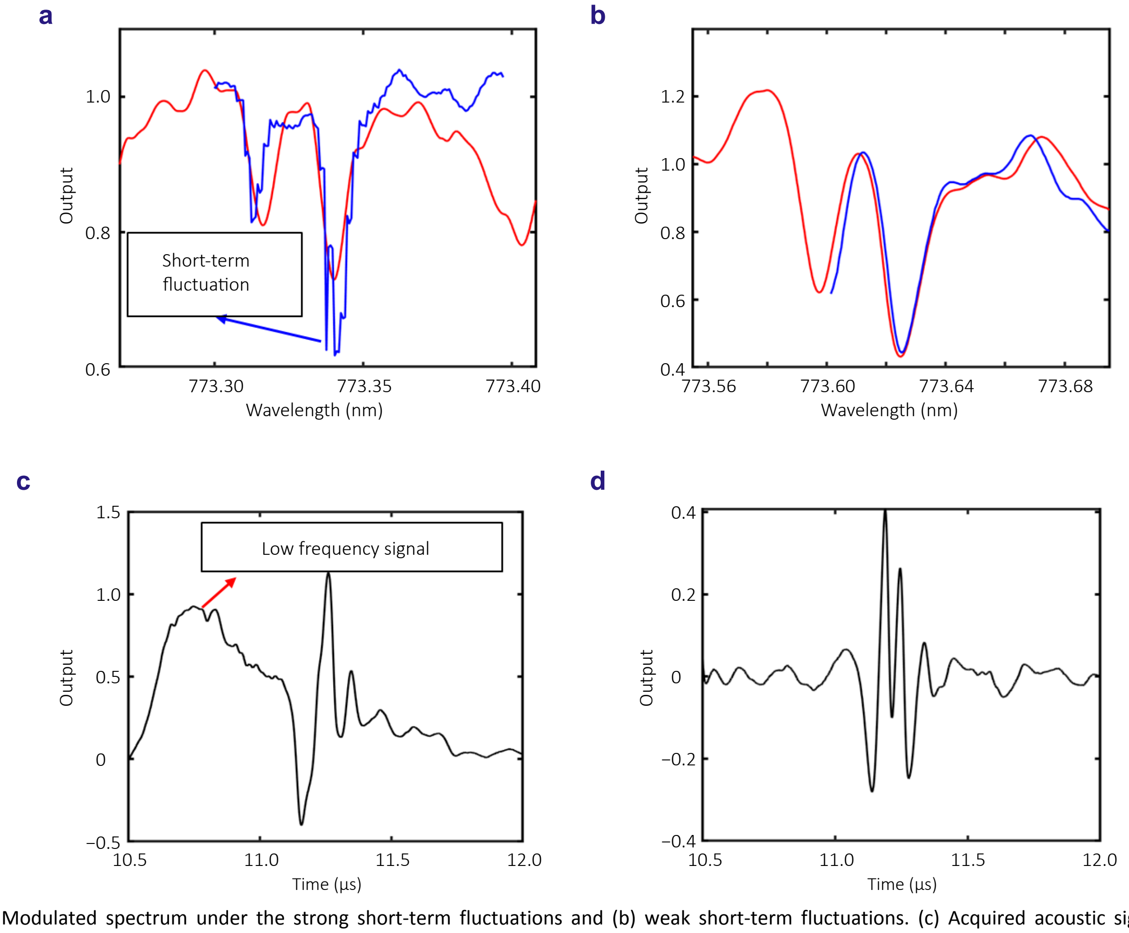

Fig. S8 | (a) Modulated spectrum under the strong short-term fluctuations and (b) weak short-term fluctuations. (c) Acquired acoustic signals from a microring with low frequency signal due to the long-term thermal transients, and (d) filtered signal.

the excitation directions such that the laser illumination on Si was indirect. Additionally, selecting an excitation wavelength outside the absorption band of Si further suppressed the transient thermal response.

Furthermore, as indicated in Fig. S8(c, d), signals acquired at the slope of the resonances exhibited low-frequency components in the time domain due to long-term thermal transients, which origin of the short-term fluctuation. These components appeared as large-amplitude, slowly varying baseline signals that interfered with photoacoustic image reconstruction. Applying a high-pass filter effectively removed these low-frequency artifacts, allowing clearer extraction of the true photoacoustic signals.

References

S1. Pan JS, Li Q, Feng YM et al. Parallel interrogation of the chalcogenide-based micro-ring sensor array for photoacoustic tomography. *Nat Commun* **14**, 3250 (2023).

S2. Lee Y, Rong QZ, Song KH et al. Theoretical and experimental study on the detection limit of the micro-ring resonator based ultrasound point detectors. *Photoacoustics* **34**, 100574 (2023).
